# Opportunities for developing therapies for rare genetic diseases: focus on gain-of-function and allostery

**DOI:** 10.1186/s13023-017-0614-4

**Published:** 2017-04-17

**Authors:** Binbin Chen, Russ B. Altman

**Affiliations:** 1 Department of Genetics, Stanford University School of Medicine, Stanford, CA USA; 2 Department of Bioengineering, Stanford University School of Medicine, Stanford, CA USA

**Keywords:** Genetic diseases, Rare diseases, Orphan drugs, Drug targets, Gain-of-function, Allosteric, Drug discovery

## Abstract

**Background:**

Advances in next generation sequencing technologies have revolutionized our ability to discover the causes of rare genetic diseases. However, developing treatments for these diseases remains challenging. In fact, when we systematically analyze the US FDA orphan drug list, we find that only 8% of rare diseases have an FDA-designated drug. Our approach leverages three primary insights: first, diseases with gain-of-function mutations and late onset are more likely to have drug options; second, drugs are more often inhibitors than activators; and third, some disease-causing proteins can be rescued by allosteric activators in diseases due to loss-of-function mutations.

**Results:**

We have developed a pipeline that combines natural language processing and human curation to mine promising targets for drug development from the Online Mendelian Inheritance in Man (OMIM) database. This pipeline targets diseases caused by well-characterized gain-of-function mutations or loss-of-function proteins with known allosteric activators. Applying this pipeline across thousands of rare genetic diseases, we discover 34 rare genetic diseases that are promising candidates for drug development.

**Conclusion:**

Our analysis has revealed uneven coverage of rare diseases in the current US FDA orphan drug space. Diseases with gain-of-function mutations or loss-of-function mutations and known allosteric activators should be prioritized for drug treatments.

**Electronic supplementary material:**

The online version of this article (doi:10.1186/s13023-017-0614-4) contains supplementary material, which is available to authorized users.

## Background

Rare diseases are defined as diseases affecting fewer than 200,000 patients in the US or fewer than 1 in 2000 people in the EU; over 25 million Americans suffer from at least one of 7000 rare diseases [1]. Due to the limited market size and cost of drug development, the development of rare disease treatments continues to be challenging for pharmaceutical companies, despite incentives created in the 1983 US Orphan Drug Act [2]. By obtaining orphan drug status, a pharmaceutical company can gain regulatory benefits including application fee waivers and extended time for market protection. Thus the US FDA orphan drug list is a barometer for the current treatment development for rare diseases. In this study, we examine the list of current and anticipated orphan drugs, and extract trends that may suggest other opportunities for development of useful therapies.

Many rare diseases have been linked to genetic abnormalities, and next-generation sequencing has accelerated our ability to make such links [3]. It took more than 10 years to identify the cystic fibrosis (MIM: 219700) causing gene *CFTR* by chromosomal walking in 1980s [4]. In contrast, with next-generation sequencing and other high throughput technologies, researchers have linked hundreds of mutations to rare diseases in the last few years [5]. The Online Mendelian Inheritance in Man (OMIM) curates both genetic and clinical information about rare diseases caused by single mutations [6]. Single mutation diseases have better understood pathological mechanisms, which is critical for drug development [7]. We therefore use the OMIM as the basis of our search for rare disease targets.

Most small molecule drugs inhibit their targets [8]. When a protein structure is altered, gain-of-function changes are more easily modulated by small molecules than loss-of-function changes; it is more difficult to rescue function. Not surprisingly, the field has had more success developing antagonists than agonists. For example, Drugbank, one of the most commonly used drug databases, includes more than 1700 small molecule inhibitors or antagonists, but only 423 small molecule activators or agonists [9].

Solved protein three-dimension (3D) structures provide a molecular basis for understanding the implications of coding variations on protein conformation, and enable rational drug design [10–12]. Thus, our study focuses on the subset of potential drug targets with both gain-of-function mutations and available 3D protein structures.

**Fig. 1:**
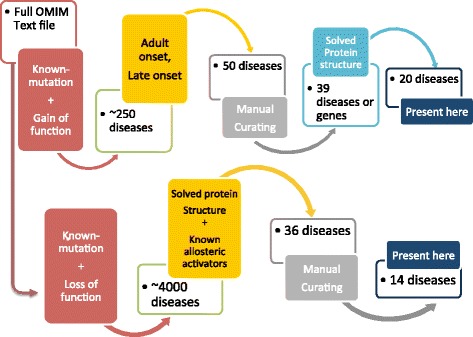
Texting mining algorithm to search for targetable rare diseases. We filtered all Mendelian diseases with known mutated genes in the OMIM for gain-of-function and late clinical onset related terms for the fist step of filtering to determine our disease targets. In a parallel branch of the pipeline, we filtered for diseases due to loss-of-function mutations with known allosteric activators. All candidates must have a solved protein structure. We manually verified the final disease list to ensure each disease mechanism and onset match our computationally generated label

In brief, we have built a pipeline (Fig. 1) to search for small molecule drug development opportunities among rare genetic diseases based on the following three assumptions. First, the disease target should be caused by a single gain-of-function mutation, so we can focus on inhibiting a single disease-driver protein rather than multiple pathways. Second, the disease should have late or adult onset, which provides a large time window to introduce therapies. Finally, the primary disease gene product should have a solved crystal structure, which is desirable for rational-based inhibitor design. Although these limit the scope of our analysis, they provide a clear rational for moving forward when the criteria are met.

Our pipeline also supports targeting diseases due to loss-of-function mutations with a known allosteric activator (Fig. 1). Allosteric regulation is a common feature in enzymatic activity. In some cases, an allosteric activator can increase the activity of a mutated enzyme, moving it towards a more physiologically normal range [13]. For example, N-carbamylglutamate (carglumic acid) can treat carbamyl phosphate synthetase I (CPSI) deficiency (MIM:237300) due to its ability to activate CPSI via an allosteric site [14] N-carbamylglutamate was approved by the FDA in 2010 [15]. The Allosteric Database (ASD) provides protein and allosteric modulator pairs that may be useful in diseases due to loss-of-function mutations [16].

## Methods

### Overview of pipeline to search for drug targets

We demonstrate the overall pipeline in the Fig. 1. We downloaded the complete OMIM database including mutated genes and disease descriptions in June 2015 [6]. Only diseases with known mutations are considered in our analysis.

First, we obtained a list of the potential diseases due to gain-of-function mutations by filtering for any OMIM disease entries mentioning gain-of-function related terms (Additional file 1: Table S1). We assumed the rest of diseases are diseases due to loss-of-function mutations. For each subset of diseases, we filtered them with our selection criteria discussed below to generate the final candidate list.

### Searching for gain-of-function rare genetic diseases

To become candidates, all diseases due to gain-of-function mutations (Additional file 1: Table S7) must have late clinical onset and their mutated proteins need to have solved crystal structures. To determine whether a disease has late onset, we required at least one related term (Additional file 1: Table S9) to appear in the OMIM disease description section. We determined crystal structure availability with the Gene ID provided by the OMIM and the UniProt mapping tool [17]. To reduce false positives, we manually verified all potential late onset diseases due to gain-of-function mutations. Structure proteins are unlikely to be inhibited by small molecule drugs given their abundance. We manually excluded diseases caused by structure proteins from the filtered disease list using the list in Additional file 1: Table S10.

### Searching for treatable rare genetic diseases due to loss-of-function mutations

For each disease due to loss-of-function mutations from the previous step (Fig. 1), we obtain its gene ID from the OMIM. We queried the Allosteric Database 2.0 (ASD) [16] for these gene IDs to check whether a small molecule can activate each disease’s mutated proteins. Using the Uniprot mapping tool, we then checked whether these gene IDs have a solved crystal structure for their gene product. In some cases, the OMIM includes weak links between mutated genes and diseases. Thus, for the final set of candidates, we manually verified strong evidence supporting the mechanism of each disease.

### Mapping the FDA orphan drug list to targeted diseases and disease categories

We obtained the list of FDA approved and designated orphan drugs from the FDA website in July 2015 [18]. The FDA provides a treatment description for each orphan drug. For example, for *alglucerase*, the treatment description is: “for replacement therapy in patients with Gaucher's disease type I.” From each of these treatment descriptions, we extracted all disease names that appear in the Comparative Toxicogenomics Database (CTD), e.g., “Gaucher's disease” (MIM: 230800). The CTD groups diseases into categories that capture high-level characteristics such as mechanism or organ system (e.g., Genetic, Musculoskeletal). We connected each orphan drug with these CTD categories through the diseases mentioned in its treatment description, and counted which categories are most often targeted. The CTD also links genetic diseases with their OMIM identifiers. We used these identifiers to connect each orphan drug with the diseases due to gain-of-function mutations discovered in the previous step.

## Results

### Orphan drugs cover a small portion of rare diseases

**Fig. 2:**
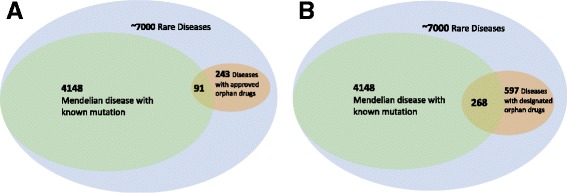
Overlap diagrams for the current approved or designated orphan drug space with the rare disease space. Each FDA approved and designated orphan drug was linked to a disease and potential OMIM ID based on the CTD table. 243 and 597 rare diseases are covered by the approved (**a**) and designed (**b**) orphan drugs. The current treatment space covers a small fraction of the rare disease space

The current FDA approved and designated orphan drugs target 243 and 597 rare disease related conditions, respectively (Fig. 2, Additional file 1: Tables S1, S2). This is still a very small portion of 7000 rare diseases [19] since only 8% of rare diseases have any designated orphan drugs.

**Fig. 3:**
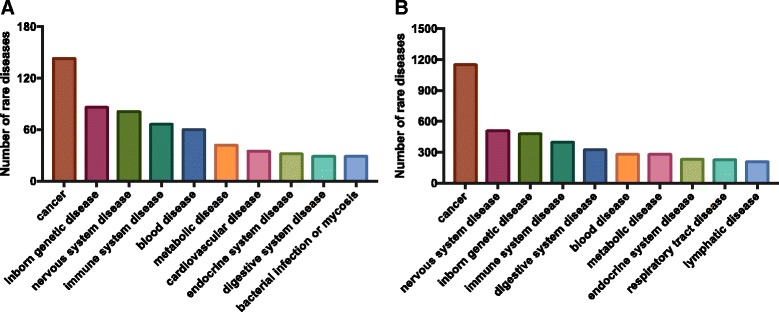
Disease category of approved or designated orphan products ranked by disease numbers. Each approved (**a**) and designated (**b**) orphan product was assigned to a disease and sequentially disease categories based on the CTD table. Numbers of diseases in the 10 most common disease category were plotted to show the distribution. Genetic diseases, the most common rare disease type, are the second most common diseases after cancer targeted by approved orphan drugs

We observe uneven coverage; cancer, neurological disorders, and genetic diseases are the most common disease categories (Fig. 3). 16–18% of the current targeted diseases are genetic diseases (Additional file 1: Tables S3, S5). Genetic diseases are underrepresented since over 60% of the known rare diseases are Mendelian diseases [6]. In comparison, 30–38% of orphan drugs target rare cancers, defined as affecting fewer than 200,000 patients (Fig. 3, Additional file 1: Tables S3, S5).

### Gain-of-functions and targetability of the disease

**Fig. 4:**
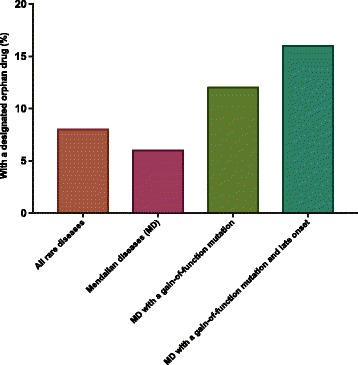
Diseases with gain-of-function mutations have higher chance to have treatment under development. Percentages of rare diseases covered by at least one FDA-designated orphan drug were plotted across categories. Mendelian diseases with a gain-of-function mutation and late clinical onset has the highest chance to be readily covered by a orphan drug (16 vs. 8% for all rare diseases)

6% of all Mendelian disorders have a designated orphan drug. In comparison, 12% of gain-of-function of Mendelian subsets have a designated orphan drug (Fig. 4). This number goes up to 16% when we further filter for adult onset diseases (Fig. 4), which supports our assumptions that diseases due to gain-of-function mutations are more readily treated with small molecules drugs.

### Promising targets

Tables 1 and 2 show the top disease candidates due to gain-of-function and loss-of-function mutations and their associated OMIM IDs and mutated genes. We group gain-of-function mutation diseases based on their molecular mechanism. No obvious pathophysiology groups are present in these diseases caused by loss-of-function mutations. Additional file 1: Tables S7 and S8 list all gain-of-function and loss-of-function mutation candidates passed our computational filter.

**Table 1:** Top targetable diseases due to gain-of-function mutations

Type	Diseases	OMIM	Gene/Protein
Over-activation	Hereditary motor and sensory neuropathy type IIC	606071	*TRPV4*
	Postsynaptic slow-channel congenital myasthenic syndrome	601462	*CHRNA1, CHRND, CHRNE, CHRNB1*
	PRPS1 superactivity	300661	*PRPS1*
	Parkinson disease-8	607060	*LRRK2*
	Tubular aggregate myopathy	160565	*STIM1*
	Achondroplasia	100800	*FGFR3*
Gene duplication	Lubs X-linked mental retardation syndrome	300260	*MECP2*
	Parkinson disease-4	605543	*SNCA*
CAG repeat	Spinocerebellar ataxia 1,2,3,6,7,17	183086, 183090, 109150, 183086, 164500, 607136	*ATXN1,2,3, CACNA1A,ATXN7, TBP*
	Huntington	6066438	*HTT*
	Spinal and bulbar muscular atrophy X-linked	313700	*AR*
Non-CAG trinucletide repeat	Friederich Ataxia 1	229300	*FXN*
	Myotonic Dystrophy 1	160900	*DMPK*
	Oculopharyngeal muscular dystrophy	164300	*PABPN1*
	Spinocerebellar ataxia 8	608768	*ATXN8*

Corresponding disease names, OMIM identifiers, and mutated genes are listed. Diseases are grouped by the molecular mechanism as indicated in the type column. The rest of potential candidates can be found in Additional file 1: Table S7

**Table 2:** Top targetable genetic diseases due to loss-of-function mutations

Disease name	OMIM	Gene/Protein	Allosteric activators
Brugada syndrome 3	611875	*CACANA1C*	BayK 8644, FPL64176
Congenital amegakaryocytic thrombocytopenia	604498	*MPL*	PF
Frontal lobe nocturnal epilepsy 3	605375	*CHRNB2*	Desformylflustrabromine and others
Glycogen storage disease V	232600	*PYGM*	AMP and IMP
Glycogen storage disease VI	232700	*PYGL*	AMP and IMP
Hereditary pancreatitis	167800	*CTRC*	BisQ, Bis Q Benzyl
Homocystinuria due to cystathionine beta-sythease deficiency	236200	*CBS*	SAM
Hyperekplexia hereditary 1	149400	*GLRA1*	Ajulemic acid, Trifluoroacetate
Isolated growth hormone deficiency type III	307200	*BTK*	PIP3
Lung cancer susceptibility	612052	*CHRNA3*	rac-12 k, rac-14e
Muscle glycogen storage disease 0	611556	*GYS1*	Glc6P
Ovarian dysgenesis 1	233300	*FSHR*	Ajulemic acid, Trifluoroacetate
Pigmented nodular adrenocortical disease	610475	*PDE11A*	Estradiol
Thrombocysthemia 2	601977	*MPL*	PF

Corresponding disease names, OMIM identifiers, mutated genes and known allosteric activators are listed. Allosteric activators are queried from the Allosteric Database. The rest of potential candidates can be found in Additional file 1: Table S8

## Discussion

Substantial opportunities and challenges remain in the current treatment development for rare genetic diseases, since 92% of rare diseases lack FDA-designated products. In 2015, 243 diseases have at least one approved orphan drug, a small increase compared to the 200 diseases reported in 2010 [2]. Our analysis shows that diseases due to gain-of-function mutations have more orphan drug designations, and thus may be good targets for drug discovery programs.

Our disease candidates for gain-of-function mutations in Table 1 include known over-activation diseases with inhibitor drug developments on trial, like PRPS1 superactivity (MIM: 300661) and achondroplasia (MIM: 100800) [20, 21]. The majority of gain-of-function disease candidates in Table 1 are neuromuscular diseases, such as spinocerebellar ataxia (MIM: 183086, 183090, 109150, 183086, 164500, 607136) or the over-activation of neuron ion channels. This is consistent with the fact that neurological disorders are one of the most common disease categories in the current FDA orphan drug list.

It is striking that over half of the diseases in Table 1 are caused by long trinucleotide repeats including both CAG and non-CAG repeats. In fact, this list contains seven out of nine known CAG repeat disorders [22]. This is because long trinucleotide repeats cause gain-of-function toxicity with late onset. Recently, drugs inhibiting the pathway involved in expressing trinucleotide repeat regions have drawn attention from the field. For example, DRB Sensitivity Inducing Factor (DSIF), which is comprised of SUPT4H1 and SUPT5H, is essential to expressing long CAG repeats in vitro and in vivo [23, 24]. A small molecule that selectively inhibits DSIF would be a potential treatment for trinucleotide repeat disorders.

A small number of Mendelian diseases with a loss-of-function mutation have intrinsic allosteric activators. Only 36 diseases passed stringent our filter, a small fraction of the 571 total diseases curated in the ASD (Fig. 1, Additional file 1: Table S8) [16]. This is because we require both an allosteric activator and a solved human protein crystal structure for the protein.

Based on FDA Rare Disease Repurposing Database [18], about 525 out of 2300 orphan drug designations in 2010 were based on drug-repurposing. Drug repurposing will continue to be an important route for orphan drug development given the substantial reduction in cost and time. Disease genes/proteins listed in the Table 1 and 2 can be evaluated against the current FDA-approved drug list to determine if any available drugs can inhibit the gain-of-function proteins or activate the loss-of-function proteins [9, 25, 26].

In this analysis we are targeting the driver mutations of rare diseases; we have not expanded our search algorithm to their upstream regulator and downstream effectors, although these are certainly worth examining [27]. The results from this study can be integrated with other rare disease research platforms like RD-Connect [28] and Orphanet [29–31] to further collaborations across disciplines and institutes.

## Conclusion

Here we present an evaluation of the current FDA orphan drug space and opportunities for treatment developments in the rare disease domain. Our pipeline provides a list of potential low-hanging fruit for orphan drug research. Drug development programs that are effective at finding inhibitors could focus on the gain-of-function candidates. Known allosteric modulators could be screened against the loss-of-function candidates to quickly evaluate the opportunities for moving forward.

## Additional file

Additional file 1: Table S1. The disease mapping for the current FDA-approved orphan drugs. **Table S2.** The disease mapping for the current FDA-designated orphan drugs. **Table S3.** The disease category mapped for the current FDA-approved orphan drugs ranked by drug number. **Table S4.** The individual diseases mapped for the current FDA-approved orphan drugs ranked by drug number. **Table S5.** The disease category mapped for the current FDA-designated orphan drugs ranked by drug number. **Table S6.** The individual diseases mapped for the current FDA-designated orphan drugs ranked by drug number. **Table S7.** The complete text-mining results of the OMIM for diseases caused by a single gain-of-function mutation. **Table S8.** The complete text-mining results of the OMIM for diseases caused by a single loss-of-function mutation and associated with a potential allosteric activator. **Table S9.** Terms used to determine gain-of-function and late disease onset in the OMIM disease description. **Table S10.** Terms used to manually determine if a gain-of-function disease is caused by a mutated structure protein. (ZIP 357 kb)

## Abbreviations

ASD: The Allosteric Database
CPSI: Carbamyl phosphate synthetase I
CTD: Comparative Toxicogenomics Database
DSIF: DRB Sensitivity Inducing Factor
MIM: OMIM ID
OMIM: Online Mendelian Inheritance in Man Database
