# Supplementary material for: Opportunities for developing therapies for rare genetic diseases: focus on gain-of-function and allostery
Source: Orphanet J Rare Dis. 2017 Apr 17;12:61. doi: 10.1186/s13023-017-0614-4 (PMC5392956; doi:10.1186/s13023-017-0614-4)
Supplement: Additional file 1: Table S1. — The disease mapping for the current FDA-approved orphan drugs. Table S2. The disease mapping for the current FDA-designated orphan drugs. Table S3. The disease category mapped for the current FDA-approved orphan drugs ranked by drug number. Table S4. The individual diseases mapped for the current FDA-approved orphan drugs ranked by drug number. Table S5. The disease category mapped for the current FDA-designated orphan drugs ranked by drug number. Table S6. The individual diseases mapped for the current FDA-designated orphan drugs ranked by drug number. Table S7. The complete text-mining results of the OMIM for diseases caused by a single gain-of-function mutation. Table S8. The complete text-mining results of the OMIM for diseases caused by a single loss-of-function mutation and associated with a potential allosteric activator. Table S9. Terms used to determine gain-of-function and late disease onset in the OMIM disease description. Table S10. Terms used to manually determine if a gain-of-function disease is caused by a mutated structure protein. (ZIP 357 kb) [file 13023_2017_614_MOESM1_ESM.zip › Supplemental data.pdf]

Supplemental Tables

(in the single excel file attached)

- Table S1. The disease mapping for the current FDA-approved orphan drugs
- Table S2. The disease mapping for the current FDA-designated orphan drugs
- Table S3. The disease category mapped for the current FDA-approved orphan drugs ranked by drug number
- Table S4. The individual diseases mapped for the current FDA-approved orphan drugs ranked by drug number
- Table S5. The disease category mapped for the current FDA-designated orphan drugs ranked by drug number
- Table S6. The individual diseases mapped for the current FDA-designated orphan drugs ranked by drug number
- Table S7. The complete text-mining results of the OMIM for diseases caused by a single gain-of-function mutation
- Table S8. The complete text-mining results of the OMIM for diseases caused by a single loss-of-function mutation and associated with a potential allosteric activator

Category	Gain-of-function	Late onset
Terms	gain-of-function<br>gain of function<br>over-activation<br>over activation<br>overexpression<br>over-expression<br>over expression	adult onset<br>adult on-set<br>adult on set<br>late onset<br>late on-set<br>late on set

Table S9. Terms used to determine gain-of-function and late disease onset in the OMIM disease description.

Category	Structure protein
Gene names	Actin, Actinin, Aggrecan, Cadherin, Collagen, Decorin, Elastin, Fibrinogen, Fibronectin, Heparan, Keratin, Laminin, Mucin, Myosin, Tropomysin, Troponin, Vimentin

Table S10. Terms used to manually determine if a gain-of-function disease is caused by a mutated structure protein
